# High Prevalence of Hepatitis C Infection Among Adult Patients at Four Urban Emergency Departments — Birmingham, Oakland, Baltimore, and Boston, 2015–2017

**DOI:** 10.15585/mmwr.mm6919a1

**Published:** 2020-05-15

**Authors:** James W. Galbraith, Erik S. Anderson, Yu-Hsiang Hsieh, Ricardo A. Franco, John P. Donnelly, Joel B. Rodgers, Elissa M. Schechter-Perkins, William W. Thompson, Noele P. Nelson, Richard E. Rothman, Douglas A.E. White

**Affiliations:** ^1^Department of Emergency Medicine, University of Mississippi Medical Center, Jackson, Mississippi; ^2^Department of Emergency Medicine, Alameda Health System–Highland Hospital, Oakland, California; ^3^Department of Emergency Medicine, Johns Hopkins School of Medicine, Baltimore, Maryland; ^4^Department of Medicine, University of Alabama School of Medicine, Birmingham, Alabama; ^5^Department of Learning Health Sciences, University of Michigan Medical School, Ann Arbor, Michigan; ^6^Institute for Healthcare Policy and Innovation, University of Michigan, Ann Arbor, Michigan; ^7^Department of Emergency Medicine, University of Alabama at Birmingham, Birmingham, Alabama; ^8^Department of Emergency Medicine, Boston University, Boston, Massachusetts; ^9^Division of Viral Hepatitis, National Center for HIV/AIDS, Viral Hepatitis, STD, and TB Prevention, CDC.

Identifying persons with hepatitis C virus (HCV) infection has become an urgent public health challenge because of increasing HCV-related morbidity and mortality, low rates of awareness among infected persons, and the advent of curative therapies (*1*). Since 2012, CDC has recommended testing of all persons born during 1945–1965 (baby boomers) for identification of chronic HCV infection (*1*); urban emergency departments (EDs) are well positioned venues for detecting HCV infection among these persons. The United States has witnessed an unprecedented opioid overdose epidemic since 2013 that derives primarily from commonly injected illicit opioids (e.g., heroin and fentanyl) (*2*). This injection drug use behavior has led to an increase in HCV infections among persons who inject drugs and heightened concern about increases in human immunodeficiency virus (HIV) and HCV infection within communities disproportionately affected by the opioid crisis (*3*,*4*). However, targeted strategies for identifying HCV infection among persons who inject drugs is challenging (*5*,*6*). During 2015–2016, EDs at the University of Alabama at Birmingham; Highland Hospital, Oakland, California; Johns Hopkins Hospital, Baltimore, Maryland; and Boston University Medical Center, Massachusetts, adopted opt-out (i.e., patients can implicitly accept or explicitly decline testing), universal hepatitis C screening for all adult patients. ED staff members offered HCV antibody (anti-HCV) screening to patients who were unaware of their status.* During similar observation periods at each site, ED staff members tested 14,252 patients and identified an overall 9.2% prevalence of positive results for anti-HCV among the adult patient population. Among the 1945–1965 birth cohort, prevalence of positive results for anti-HCV (13.9%) was significantly higher among non-Hispanic blacks (blacks) (16.0%) than among non-Hispanic whites (whites) (12.2%) (p<0.001). Among persons born after 1965, overall prevalence of positive results for anti-HCV was 6.7% and was significantly higher among whites (15.3%) than among blacks (3.2%) (p<0.001). These findings highlight age-associated differences in racial/ethnic prevalences and the potential for ED venues and opt-out, universal testing strategies to improve HCV infection awareness and surveillance for hard-to-reach populations. This opt-out, universal testing approach is supported by new recommendations for hepatitis C screening at least once in a lifetime for all adults aged ≥18 years, except in settings where the prevalence of positive results for HCV infection is <0.1% (*7*).

A retrospective study from four urban academic EDs located in Birmingham, Alabama; Oakland, California; Boston, Massachusetts; and Baltimore, Maryland was conducted with approval from each institution’s local Institutional Review Board. Each ED implemented opt-out, universal hepatitis C testing at different times and using differing methodologies among patients who reported no history of HCV infection. The period of observation for this study was 4 months, starting 1 month after initial implementation of opt-out, universal hepatitis C screening. Because of programmatic changes during the observation period at Johns Hopkins ED, only 3 months of observation is reported. All sites used the Abbott Architect anti-HCV assay (Abbott Diagnostics) for testing, with results available during the ED visit, and reflex HCV RNA testing performed on specimens collected during the ED encounter from persons with anti-HCV positive results. Each site used dedicated linkage-to-care coordinators to deliver positive test results and facilitate referral to HCV infection care.

ED sites collected cumulative hepatitis C testing outcomes for the 4-month study period, including cumulative anti-HCV results stratified by birth year, race/ethnicity, sex, and insurance type. Deidentified data were collected for aggregation and analysis at the University of Alabama at Birmingham site. Patient characteristics and prevalence estimates for positive results for anti-HCV were reported with 95% confidence intervals across sites. P-values <0.05 were considered statistically significant. STATA (version 15.1; StataCorp) was used to conduct all statistical analyses.

Using opt-out, universal hepatitis C screening (Table 1), EDs performed a total of 14,252 tests on unique visitors, and 1,315 (9.2%) had positive test results for anti-HCV (Table 2). HCV RNA testing for current infection was performed for 1,118 (85%) visitors with positive test results for anti-HCV, and 693 (62%) of these persons had positive HCV RNA test results, indicating current HCV infection. The prevalence of positive results for anti-HCV was higher among persons in the 1945–1965 birth cohort (13.9%) than among those in the cohort born after 1965 (6.7%); however, the younger cohort accounted for 47.8% (628 of 1,315) of total cases reactive to anti-HCV identified. 

**TABLE 1:** Universal hepatitis C testing programs at four urban emergency departments (EDs) — Birmingham, Alabama; Oakland, California; Baltimore, Maryland; and Boston, Massachusetts, 2015–2017

Study site	Study dates	Program overview
University of Alabama at Birmingham Hospital, Birmingham, Alabama	Oct 15, 2015–Feb 15, 2016	Opt-out, nurse-driven intervention using electronic EHR prompts, physician counseling for positive results for anti-HCV during ED visit, or specimens for HCV RNA testing collected during visit for persons with positive results for anti-HCV
Highland Hospital, Oakland, California	Oct 15, 2015–Feb 15, 2016	Opt-out, nurse-driven intervention using EHR prompts at triage, physician counseling for positive results for anti-HCV during ED visit, or specimens for HCV RNA testing collected during visit for persons with positive results for anti-HCV
Johns Hopkins Hospital, Baltimore, Maryland	May 1, 2016–Jul 31, 2016*	Opt-out, triage nurse-driven intervention using EHR prompts, HCV program staff members informing and consulting positive result for anti-HCV at callback after ED visit, or diagnostic HCV RNA testing at callback after the visit for persons with positive results for anti-HCV
Boston University Medical Center, Boston, Massachusetts	Nov 2, 2016–Feb 28, 2017	Opt-out, EHR-driven intervention using an EHR clinical decision support tool for all ED patients undergoing phlebotomy, with reflex HCV RNA testing for persons with positive results for anti-HCV

**Abbreviations:** anti-HCV = HCV antibody; EHR = electronic health record; HCV = hepatitis C virus.

* Limited to a 3-month testing period because of programmatic changes occurring during the observation period.

**TABLE 2:** Universal hepatitis C testing results at four urban emergency departments (EDs) — Birmingham, Alabama; Oakland, California; Baltimore, Maryland; and Boston, Massachusetts, 2015–2017

Client and testing characteristic	Study sites and dates				
	University of Alabama at Birmingham Hospital, Birmingham, Alabama Oct 15, 2015–Feb 15, 2016	Highland Hospital, Oakland, California Oct 15, 2015–Feb 15, 2016	Johns Hopkins Hospital, Baltimore, Maryland May 1, 2016–Jul 31, 2016*	Boston University Medical Center, Boston, Massachusetts Nov 2, 2016–Feb 28, 2017	All sites
Unique ED visitors	18,916	18,272	13,069	26,870	77,127
Patients eligible for hepatitis C testing	13,999	9,585	7,639	12,284	43,507^†^
Anti-HCV tests performed	5,973	2,900	1,638	3,741	14,252^§^
Total anti-HCV positive tests (%)	459 (7.7)	166 (5.7)	120 (7.3)	570 (15.2)	1,315 (9.2)
Adults born 1945–1965, positive test results for anti-HCV/anti-HCV tests (%)	232/2,205 (10.5)	98/713 (13.7)	69/437 (15.8)	288/1,585 (18.2)	687/4,940 (13.9)
Born after 1965, positive test results for anti-HCV/anti-HCV tests (%)	227/3,768 (6.0)	68/2,187 (3.1)	51/1,201 (4.2%)	282/2,156 (13.1)	628/9,312 (6.7)
Total HCV RNA tests performed (%)	398 (86.9)	125 (75.3)	38 (31.6)	557 (97.7)	1,118 (85)
Total current HCV infections (positive test results for HCV RNA) (%)	252 (63.3)	79 (63.2)	27 (71.1)	335 (60.1)	693 (62.0)
Estimated prevalence of positive results for HCV RNA (%)	4.9	3.6	5.2	9.1	5.7
State and national estimated prevalence of positive results for HCV RNA, %	Alabama, 0.85	California, 1.25	Maryland, 1.00	Massachusetts, 0.85	National, 0.93

**Abbreviations:** anti-HCV = HCV antibody; EHR = electronic health record; HCV = hepatitis C virus.

* Limited to a 3-month testing period because of programmatic changes occurring during the observation period.

^†^ Born after 1944, aged ≥18 years, medically or surgically stable, and no self-reported history of prior HCV infection.

^§^ Reasons testing not performed included that the patient declined testing or venipuncture was not performed because no diagnostic tests requiring venipuncture were ordered by the ED provider.

Significant differences in positive results for anti-HCV by birth cohort and race/ethnicity were identified (Table 3). Among persons born during 1945–1965, overall positive results for anti-HCV prevalence was significantly higher among blacks (16.0%) than among whites (12.2%) (p<0.001). In contrast, overall prevalence of positive results for anti-HCV among persons born after 1965 was higher among whites (15.3%) than among blacks (3.2%) (p<0.001). Significant differences in positive results for anti-HCV were identified among ED sites regarding race/ethnicity for both birth cohorts. Positive results for anti-HCV among whites born after 1965 was higher among patients evaluated at the University of Alabama at Birmingham (11.7%), Johns Hopkins (11.8%), and Boston University (30.1%) sites than among those evaluated at Highland Hospital (3.2%).

**TABLE 3:** Prevalence of positive results for hepatitis C virus antibody (anti-HCV) and prevalence differences, by study site and patient characteristics — Birmingham, Alabama; Oakland, California; Baltimore, Maryland; and Boston, Massachusetts, 2015–2017

Characteristic	All sites		University of Alabama at Birmingham Hospital, Birmingham, Alabama		Highland Hospital, Oakland, California		Johns Hopkins Hospital, Baltimore, Maryland		Boston University Medical Center, Boston, Massachusetts	
	Total no. (% positive test results for anti-HCV)	Prevalence difference (95% CI)*	Total no. (% positive test results for anti-HCV)	Prevalence difference (95% CI)*	Total no. (% positive test results for anti-HCV)	Prevalence difference (95% CI)*	Total no. (% positive test results for anti-HCV)	Prevalence difference (95% CI)*	Total no. (% positive test results for anti-HCV)	Prevalence difference (95% CI)*
**Born during 1945–1965**										
**Sex**										
Women	2,325 (8.3)	Referent	1,100 (6.2)	Referent	298 (10.1)	Referent	190 (7.9)	Referent	737 (11.0)	Referent
Men	2,615 (18.9)	10.5 (8.6 to 12.4)	1,105 (14.8)	8.7 (6.3 to 11.2)	415 (16.4)	6.3 (1.3 to 11.9)	247 (21.9)	14.0 (8.2 to 20.9)	848 (24.4)	13.4 (9.7 to 16.7)
**Race/Ethnicity**										
White, NH	1,695 (12.2)	−3.8 (−5.8 to 1.6)	1,058 (9.5)	−2.4 (−5.0 to 0.4)	92 (13.0)	−4.3 (−11.1 to 5.2)	121 (3.3)	−19.2 (−24.8 to 13.6)	424 (21.2)	2.5 (−2.1 to 7.2)
Black, NH	2,534 (16.0)	Referent	1,093 (11.8)	Referent	358 (17.3)	Referent	284 (22.5)	Referent	799 (18.8)	Referent
Other/Missing	711 (10.7)	−5.3 (−7.9 to −2.5)	54 (5.6)	−6.2 (−11.1 to 1.4)	263 (9.1)	−8.2 (−13.3 to −2.4)	32 (3.1)	−19.4 (−26.0 to −10.9)	362 (13.3)	−5.5 (−9.5 to −0.8)
**Insurance type**										
Commercial	1,138 (8.4)	−9.3 (−11.8 to −7.2)	562 (4.8)	−12.1 (−16.1 to −8.1)	23 (13.0)	0.2 (−11.7 to 19.8)	269 (11.9)	−15.6 (−30.4 to 1.4)	284 (12.0)	−8.7 (−13.5 to −3.8)
Medicare	1,482 (13.6)	−4.1 (−6.7 to −1.8)	844 (9.5)	−7.4 (−11.6 to −3.4)	115 (19.1)	6.3 (−1.8 to 14.1)	79 (19.0)	−8.5 (−26.6 to 6.8)	444 (19.1)	−1.5 (−6.1 to 3.0)
Medicaid/Publicly funded	1,702 (17.7)	Referent	420 (16.9)	Referent	467 (12.9)	Referent	40 (27.5)	Referent	775 (20.7)	Referent
Other/Missing	618 (14.1)	−3.7 (−6.9 to −0.2)	379 (14.3)	−2.7 (−7.5 to 2.7)	108 (12.0)	−0.8 (−7.6 to 6.5)	49 (22.5)	−5.1 (−23.9 to 13.0)	82 (11.0)	−9.7 (−16.9 to −1.8)
**Born after 1965**										
**Sex**										
Women	5,119 (5.1)	Referent	2,149 (4.1)	Referent	1,121 (2.8)	Referent	680 (3.5)	Referent	1,169 (10.2)	Referent
Men	4,193 (8.7)	3.6 (2.5 to 4.7)	1,619 (8.5)	4.4 (2.8 to 6.0)	1,066 (3.5)	0.7 (−0.7 to 2.2)	521 (5.2)	1.7 (−0.6 to 4.0)	987 (16.5)	6.3 (3.6 to 9.5)
**Race/Ethnicity**										
White, NH	2,623 (15.3)	12.2 (10.6 to 13.6)	1,554 (11.7)	9.7 (8.1 to 11.6)	185 (3.2)	−0.2 (−2.8 to 2.4)	280 (11.8)	9.7 (6.1 to 13.8)	604 (30.1)	23.9 (19.9 to 27.7)
Black, NH	4,711 (3.2)	Referent	2,063 (2.0)	Referent	867 (3.5)	Referent	780 (2.1)	Referent	1,001 (6.2)	Referent
Other/Missing	1,978 (3.9)	0.7 (−0.2 to 1.7)	151 (3.3)	1.3 (−1.0 to 5.0)	1,135 (2.8)	−0.6 (−2.4 to 7.6)	141 (1.4)	−0.6 (−2.3 to 2.2)	551 (6.9)	0.7 (−1.8 to 3.5)
**Insurance type**										
Commercial	2,370 (3.0)	−5.6 (−6.8 to −4.5)	1,065 (2.2)	−3.0 (−4.7 to −1.3)	94 (3.2)	−0.0 (−3.0 to 4.1)	800 (3.4)	−7.0 (−13.0 to −2.1)	411 (4.4)	−12.1 (−15.2 to −9.5)
Medicare	634 (9.0)	0.4 (−1.8 to 2.8)	359 (6.4)	1.3 (−1.5 to 4.3)	48 (4.2)	0.9 (−3.6 to 8.3)	57 (1.8)	−8.6 (−15.3 to −2.0)	170 (18.2)	1.7 (−3.7 to 8.7)
Medicaid/Publicly funded	3,944 (8.6)	Referent	935 (5.1)	Referent	1,486 (3.2)	Referent	135 (10.4)	Referent	1,388 (16.5)	Referent
Other/Missing	2,364 (6.8)	−1.8 (−3.1 to −0.4)	1,409 (9.4)	4.3 (2.2 to 6.5)	559 (2.7)	−0.5 (−2.0 to 1.2)	209 (4.3)	−6.1 (−12.4 to −0.9)	187 (2.1)	−14.4 (−16.9 to −11.5)

**Abbreviations:** CI = confidence interval, NH = non-Hispanic.

* Bias-corrected 95% CIs for prevalence differences calculated by using 1,000 bootstrap replicates.

Among persons born during 1945–1965, and those born after 1965, prevalence of positive results for anti-HCV was significantly higher among men (18.9% and 8.7%, respectively), than among women (8.3% and 5.1%, respectively) (p<0.001). No statistically significant differences were identified in positive results for anti-HCV by sex among ED sites for either birth cohort (Table 3).

Prevalence of positive results for anti-HCV was higher among Medicaid or other public insurance recipients, persons with other or missing insurance information, and Medicare recipients, than among commercially insured persons in both the 1945–1965 birth cohort (17.7%, 14.1%, and 13.6%, respectively, versus 8.4%; p<0.001) and persons born after 1965 (8.6%, 6.8%, and 9.0%, respectively, versus 3.0%; p<0.001).

## Discussion

Opt-out, universal HCV screening in four geographically diverse, urban EDs identified a high prevalence of previously unrecognized positive results for anti-HCV in approximately one of every 11 (9.2%) adult patients tested. Prevalence of positive results for HCV RNA at the combined ED sites was 5.7%, which was substantially higher than the estimated overall U.S. prevalence of positive results for HCV RNA of 0.95% (*8*). At the state level, ED prevalence of positive results for HCV RNA ranged from three to fivefold higher than the upper-estimated prevalence of positive results for HCV RNA rates in each respective state (*8*). These findings demonstrate the high yield and potential impact of an ED-based opt-out, universal testing strategy.

Considering that the advent of HCV curative therapies, potential exists to eliminate HCV infection from U.S. communities. For this reason, identification of persons unaware of their HCV infection has become a public health priority. Because of the increasing incidence of HCV infection among persons who inject drugs, testing and treatment of this population is needed for both infection prevention and for ending the HCV infection epidemic. Although recent studies of ED-based, targeted hepatitis C testing have highlighted the high prevalence of positive results for anti-HCV among the 1945–1965 birth cohort (10.3%–11.6%), ED-based programs have been challenged to systematically identify and test an increasing number of younger persons who inject drugs (*5*,*6*,*9*,*10*).

Although three quarters of HCV infections in the United States are among persons born during 1945–1965, this study demonstrates that nearly half of all persons reactive to anti-HCV identified in EDs were among the cohort born after 1965. This finding is consistent with two recent ED studies, both of which reported that an ED-based 1945–1965 birth cohort strategy alone would fail to identify half of persons with HCV infection (*8*,*9*). Most striking in the current study was the high prevalence of positive results for anti-HCV (6.7%) noted among the younger population, driven by the high prevalence of positive results for anti-HCV among whites (15.3%). Although behavioral risk factors could not be confirmed for this study, this racial/ethnic difference is consistent with the epidemiology of HCV infection and injection drug use behavior (*2*).

By leveraging lessons learned from national HIV testing efforts, opt-out, universal HCV screening might improve rates of hepatitis C testing among populations at high risk by reducing patient and provider stigma associated with identification of hepatitis C behavioral risks as a prerequisite for testing. In addition, the opt-out, universal screening strategy that requires less risk behavior questioning is easier to operationalize in EDs challenged by competing priorities.

Although both targeted and opt-out, universal ED-based hepatitis C testing strategies are effective at identifying previously unrecognized HCV infections, reimbursement for testing and challenging HCV infection care navigation remain crucial barriers. A 2014 decision from the U.S. Department of Health and Human Services and Centers for Medicare & Medicaid Services precluding EDs from reimbursement for hepatitis C testing might be limiting adoption of any systematic hepatitis C testing in the majority of EDs.^†^ In addition, the high number of persons with HCV infection identified in the ED setting challenges HCV navigation programs and requires robust support to effectively direct persons who test positive to HCV treatment and other necessary health services, including primary care, social services, and substance use treatment.

The findings in this study are subject to at least three limitations. First, identifying previously unrecognized HCV infection is limited by the patient’s recall of their prior HCV infection history and is therefore subject to bias. Second, 29,255 persons identified as being eligible for hepatitis C testing in the study EDs were not tested because a venipuncture was not performed for other diagnostics ordered by the ED provider during the visit, a prior HCV test result was identified in the electronic health record, or the patient declined to be tested. This is consistent with previously reported findings from ED-based targeted hepatitis C testing (*5*,*6*), and bias was not introduced toward testing persons appearing to be at high risk. Finally, study findings are limited to four geographically diverse, urban academic EDs, and might not apply to all U.S. geographic areas or in nonurban or community EDs.

The high prevalence of HCV infection identified among persons born after 1965 as well as those born during 1945–1965 supports continued assessment of ED-based hepatitis C testing, as well as an opt-out, universal screening strategy among similar high-prevalence health care venues. Given the high prevalence of positive results for HCV RNA identified among a younger, predominately white cohort known to be disproportionately affected by the opioid crisis, ED-based opt-out, universal HCV screening might play an important role in surveillance and combat of interrelated epidemics of opioid overdose and bloodborne viral infections through harm-reduction interventions and navigation to HCV treatment.

**Summary**

**What is already known about this topic?**

Targeted testing for hepatitis C virus (HCV) infection in emergency departments (EDs) has been demonstrated to be a high-yield and effective intervention for identifying previously unrecognized infections, especially among persons born during 1945–1965.

**What is added by this report?**

Opt-out, universal HCV screening in EDs identified that nearly half (47.5%) of infections were among persons born after 1965.

**What are the implications for public health practice?**

Opt-out, universal screening in EDs can identify a larger number of previously unrecognized HCV infections, especially among persons born after 1965. ED-based opt-out, universal hepatitis C screening can be vital in combating and surveilling the interrelated epidemics of opioid overdose and bloodborne viral infections through harm-reduction interventions and navigation to HCV treatment.

## Footnotes

* To reduce potential duplicate testing of patients, sites utilized electronic health record mechanisms to identify and cancel HCV antibody orders on persons with prior HCV antibody testing in the last year, as well as any prior positive anti-HCV or RNA result.

† https://www.cms.gov/medicare-coverage-database/details/nca-decision-memo.aspx?NCAId=272.

## References

[1] Smith BD, Morgan RL, Beckett GA, . Recommendations for the identification of chronic hepatitis C virus infection among persons born during 1945–1965. MMWR Recomm Rep 2012;61(No. RR-4). 22895429

[2] Scholl L, Seth P, Kariisa M, Wilson N, Baldwin G. Drug and opioid-involved overdose deaths—United States, 2013–2017. MMWR Morb Mortal Wkly Rep 2018;67:1419–27. 10.15585/mmwr.mm675152e1 30605448 PMC6334822

[3] Suryaprasad AG, White JZ, Xu F, Emerging epidemic of hepatitis C virus infections among young nonurban persons who inject drugs in the United States, 2006–2012. Clin Infect Dis 2014;59:1411–9. 10.1093/cid/ciu643 25114031

[4] Van Handel MM, Rose CE, Hallisey EJ, County-level vulnerability assessment for rapid dissemination of HIV or HCV infections among persons who inject drugs, United States. J Acquir Immune Defic Syndr 2016;73:323–31. 10.1097/QAI.0000000000001098 27763996 PMC5479631

[5] Galbraith JW, Franco RA, Donnelly JP, Unrecognized chronic hepatitis C virus infection among baby boomers in the emergency department. Hepatology 2015;61:776–82. 10.1002/hep.27410 25179527

[6] White DAE, Anderson ES, Pfeil SK, Trivedi TK, Alter HJ. Results of a rapid hepatitis C virus screening and diagnostic testing program in an urban emergency department. Ann Emerg Med 2016;67:119–28. 10.1016/j.annemergmed.2015.06.023 26253712

[7] Schillie S, Wester C, Osborne M, Wesolowski L, Ryerson AB. CDC recommendations for hepatitis C screening among adults—United States, 2020. MMWR Recomm Rep 2020;69(No. RR-2). 10.15585/mmwr.rr6902a1 32271723 PMC7147910

[8] Rosenberg ES, Rosenthal EM, Hall EW, Prevalence of hepatitis C virus infection in US states and the District of Columbia, 2013 to 2016. JAMA Netw Open 2018;1:e186371–14. 10.1001/jamanetworkopen.2018.6371 30646319 PMC6324373

[9] Merchant RC, Baird JR, Liu T, Taylor LE. HCV among The Miriam Hospital and Rhode Island Hospital adult ED patients. R I Med J (2013) 2014;97:35–9. 24983020 PMC4349365

[10] Hsieh Y-H, Rothman RE, Laeyendecker OB, Evaluation of the Centers for Disease Control and Prevention recommendations for hepatitis C virus testing in an urban emergency department. Clin Infect Dis 2016;62:1059–65. 10.1093/cid/ciw074 26908800 PMC4826455

